# Public perspective on potential treatment intervention harm in clinical trials—terminology and communication

**DOI:** 10.1186/s13063-024-08418-w

**Published:** 2024-08-31

**Authors:** Rachel Phillips, Dongquan Bi, Beatriz Goulão, Marie Miller, Malak El-Askary, Oluyemi Fagbemi, Curie Freeborn, Maria Giammetta, Noura El Masri, Peter Flockhart, Manos Kumar, Mike Melvin, Dianne Murray, Anthony Myhill, Laila Saeid, Shanice Thomas, Graeme MacLennan, Victoria Cornelius

**Affiliations:** 1 Imperial Clinical Trials Unit, School of Public Health, Imperial College London, London, UK; 2 Pragmatic Clinical Trials Unit, Centre for Evaluation and Methods, Queen Mary University of London, London, UK; 3 Health Services Research Unit, University of Aberdeen, Aberdeen, UK; 4 Public Partner, London, UK

**Keywords:** Clinical trials, Adverse events, Harms, Public partners, Patient and public involvement

## Abstract

**Background:**

Randomised controlled trials (RCTs) are typically designed to determine beneficial intervention effects. In addition, an important aspect of every trial is to collect data on any potential harmful effects, with the aim of ensuring that the benefit-risk balance is appropriate. The language used by trialists to describe these potential harmful effects is inconsistent. In pharmacological trials, researchers collect adverse events; when a causal relationship is suspected adverse events are further classified as adverse reactions. Academic researchers have moved to collectively refer to these as *harm* outcomes; the pharmaceutical industry refer to these events as *safety* outcomes. In trials of complex interventions, phrases such as *unintended consequences* or *effects* are used. With the inconsistent use of terminology by researchers and the potential benefits to be gained from harmonising communications, we sought public opinion on terminology used to describe harmful effects and how these outcomes are communicated in the scientific literature, as well as in public facing material on medications.

**Methods:**

We held two in-person public involvement meetings with public partners, in London and Aberdeen in 2023. Both meetings followed a pre-specified format. We provided a background to the topic including the information researchers collect on potential harms in clinical trials and shared examples on how this information gets presented in practice. We then discussed public partners’ perspectives on terminology used and communication of intervention harm in academic journals and in public facing materials. A summary of these discussions and the main topics raised by public partners are presented.

**Results:**

Public partners endorsed the use of different terms for different situations, preferring the use of ‘side-effect’ across all contexts and reserving the use of ‘harm’ to indicate more severe events. Generally, public partners were happy with the type of information presented in public facing materials but discussions revealed that presentation of information on public NHS websites led to misconceptions about harm.

**Conclusion:**

This work provides a starting point on preferred terminology by patients and the public to describe potential harmful intervention effects. Whilst researchers have tried to seek agreement, public partners endorsed use of different terms for different situations. We highlight some key areas for improvement in public facing materials that are necessary to avoid miscommunication and incorrect perception of harm.

## Introduction

Whilst randomised controlled trials (RCTs) are designed to determine beneficial intervention effects, an important aspect of every trial is to also collect data on any potential harmful effects, with the aim of ensuring that the benefit-risk balance is appropriate. In the pharmacological setting, this is a well-established requirement of the regulator to ensure drugs are ‘safe-enough’ for their intended use, i.e. to ensure harms do not outweigh the potential benefits, and the information collected is used to inform product labels [1–3]. Whilst trials of complex interventions have not benefited from the same safety regulatory requirements, there is growing awareness of the importance of collecting potential unintended consequences. A leading example is the Recording HArms in Behavioural change Intervention Trials (RHABIT) initiative led by researchers from the University of Sheffield who are working on improving the recording of harms in trials of behavioural change interventions, and guidelines for recording harm in trials of digital interventions are in development at the University of Nottingham [4, 5].

Trialists typically collect untoward events (medical or other) that occur during a trial (sometimes called emerging events) and in some trials they will also predefine events of interest based on prior experience. The language used by trialists to describe these untoward or ‘harmful’ events is inconsistent and sometimes treatments have unexpected effects that are beneficial (e.g. the early trials of sildenafil (commonly known by its brand name, Viagra) to treat high blood pressure and angina pectoris where participants reported side-effects of erections, and subsequent research demonstrated its effectiveness to treat erectile dysfunction) [6]. In pharmacological trials to monitor for harm, researchers collect adverse events, which are defined as ‘*any untoward medical occurrence in a patient or clinical investigation subject administered a pharmaceutical product and which does not necessarily have to have a causal relationship with this treatment*’ [2]. When a causal relationship is suspected, adverse events are further classified as adverse reactions, defined as ‘*all noxious and unintended responses to a medicinal product related to any dose should be considered adverse drug reactions*’ [2]. Directed by a 2004 CONSORT extension, academic researchers have moved to collectively refer to these outcomes as *harm* outcomes [7]. Research from the pharmaceutical industry continues to collectively refer to these events as *safety* outcomes. In trials of complex interventions, phrases such as *unintended consequences* or *effects* are also sometimes used [8]. The language used is important as it can convey a strong message about treatment effects. For example, the use of ‘safety’ may overly reassure, where ‘harm’ may overly alarm [7, 9]. The variation in language used is a barrier to communication of results to both the public and researchers. Consistency in terminology is important as it provides clarity to aid understanding and interpretation of results.

Another complex issue is the amount and nature of the data collected within a trial on potential harmful effects. At the trial design stage, it is not possible or appropriate to specify all adverse events of interest as researchers do not know what or how many events will occur. As these events cannot be pre-specified, a range of information is collected, for example the number of recurrences, the severity of the event, how long it lasted and when it occurred. However, in journal articles reporting the main results of RCTs, information on adverse events is often simplified and presented in summary tables, where the information reported is often limited to the number of participants experiencing each event at least once [10, 11]. Results presented in journal articles are one source of data that are used to inform patient treatment options. They also inform the information that is included in product labels and that gets presented in public facing sources such as the National Health Service (NHS) Medicines A-Z website and National Institute for Health and Care Excellence’s (NICE) British National Formulary (BNF), a trusted resource for prescribers, pharmacists and other healthcare professionals in the UK which contains information about the use of medicines [12, 13].

With the inconsistent use of terminology by researchers over many years and the potential benefits to be gained from harmonising communications, we sought public opinion on terminology used to describe harmful effects and how these outcomes are communicated in the scientific literature, as well as in public facing sources such as the NHS and BNF websites. The public perspective on terminology could influence language used by trialists going forward. Communication of treatment adverse events has also been identified as a priority in public involvement in statistics in trials [14].

## Objectives

i. To discuss with public partners and seek their opinions on the terminology used to describe information on intervention harm in clinical trials.
ii. To explore public partners’ perspectives on how information on intervention harm is communicated in academic journals and in public facing materials.

## Methods

We undertook two in-person public involvement meetings with public partners, one in London (referred to as group 1) and one in Aberdeen (referred to as group 2) to ensure a broad spectrum of opinions was sought and to examine replication in the opinions shared. The opportunity to participate in the London meeting was advertised in a local (north-west London) community newsletter and on the NIHR People in Research involvement website. Twenty-seven people responded to the advert and completed a brief demographics questionnaire (capturing age, ethnicity, education and employment backgrounds), which helped ensure diversity within the group. To select group members prior to the meeting, RP and DB spoke with interested members in early August 2023 to briefly describe the project. To ensure a wide range of views would be collected, we also used these conversations to gauge interest in healthcare research, prior experience with research (whether as part of a PPI group or research participant), a willingness to share opinions and be comfortable doing so verbally in a group setting. Six people were invited to participate, including three people who had previously participated in a clinical trial, one of which had also contributed to PPI groups, and a further two people who had experience of health-based focus groups. The group took place in late August 2023 was facilitated by RP, with input from VC, DB and MM. The second meeting involved an established PPI group who work closely with the Health Services Research Unit (HSRU) at the University of Aberdeen. The meeting was advertised directly to this group and six members expressed an interest and were invited to attend, including two new members. The meeting took place in November 2023 and was facilitated by BG, with input from GM and RP.

The first meeting held in London comprised of members from a range of ages (20–64 years), ethnicities, employment statuses and educational backgrounds but was predominantly female (5/6 participants). The second meeting held in Aberdeen included members who ranged in age from 25 to 65+ years, came from a range of ethnicities, employment statuses and educational backgrounds and had an even split of female to male members.

Both meetings followed a pre-specified format lasting 2 h. The facilitators introduced the topic, provided context and shared examples to facilitate discussions on the two main objectives of the meeting: terminology and presentation of adverse event results in publications and public facing materials. Further details on discussion topics are provided below.

Examples of results presented in journal articles were taken from two published NEJM articles from March 2021 and August 2023. These were selected as examples as they had been published in a high impact journal where researchers might expect articles to present best practice and described trials of treatments for two common conditions, which we expected public partners to be aware of (obesity and depression). Example structure of the tables of adverse events that were presented at the meetings can be found in Tables 1 and 2. The data presented to the groups are shown in Table 3 of article [15], which shows a table of adverse events listed in alphabetical order with the number of participants and percentages for each event by treatment arm, and Table 3 of article [16], which shows a table of adverse events that occurred in 10% or more of participants in either treatment arm and includes number of participants and percentages, number of events and events per 100 person years for each event by treatment arm.

**Table 1:** Template adverse event table based on Table 3 of [15]

**Adverse event**	Intervention group (*N* = xx)	Control group (*N* = xx)
	*Number (percent)*	
≥1 Nonserious event		
Abdominal pain		
Acne		
Anaemia		
…		
Other

**Table 2:** Template adverse event table based on Table 3 of [16]

**Adverse event**	Intervention group (*N* = xx)			Control group (*N* = xx)		
	*No. of participants (%)*	*No. of events*	*Events/100 person-year*	*No. of participants (%)*	*No. of events*	*Events/100 person-year*
Any adverse event						
Serious adverse event						
Adverse events leading to discontinuation of treatment						
Fatal events						
Adverse events reported in ≥10% of participants						
Nausea						
Diarrhoea						
….						

The public facing materials were chosen based on the interventions used in these journal articles. Examples used in the meeting were taken from the NHS website on side-effects and can be found here https://www.nhs.uk/medicines/escitalopram/side-effects-of-escitalopram/ and from the side-effects section of this page https://bnf.nice.org.uk/drugs/semaglutide/ on the NICE BNF website. The NHS website lists events in bullet points, starting with common side-effects (those in more than 1 in 100), followed by the serious side-effects, serious allergic reactions and sexual side-effects, finishing with an instruction to see the leaflet in the medicine packet for other effects and to report any suspected side-effects to the Medicines and Healthcare products Regulatory Agency’s (MHRA) Yellow Card scheme (https://yellowcard.mhra.gov.uk/). The BNF contains information on drug action and indications for use before listing side-effects. It starts with general side-effects that are listed in a paragraph by common/very common or uncommon, rare/very rare (no definition), then lists specific side-effects by frequency.

Whilst the format and content of the meetings were identical, the lead facilitator and wider research group in attendance differed. RP facilitated the first meeting and attended the second meeting to ensure consistency and to allow an overview of both meetings to be presented. The change in lead facilitator was with the intention that any bias from knowledge of the conversations in the first meeting had limited impact on the second meeting.

Meetings were not recorded, but one researcher took notes at each meeting. Notes were organised by the assigned note taker with input from the facilitator according to the main topics raised by public partners. Assimilation of topics across both meetings was undertaken by RP and a summary of the discussions and the main topics raised by public partners were prepared and reviewed by researchers and public partners.

Topics explored across the meetings included:

1. Current terminology in use

Terms discussed were adverse events, harms, safety, side-effects and unintended consequences or effects.

Questions posed to the public partners:

i. What do these terms mean to you?
ii. Have you heard of any others?
iii. What term do you prefer?
iv. Does this change dependent on context, e.g. when the doctor talks to you and when you’re reading drug inserts?

2. How and what information is collected and how it is communicated

We explained the concepts and gave examples of recurrence, severity, seriousness, duration, relatedness and resolution. We demonstrated several ways this information could be collected, including clinician prompt, self-report and a questionnaire. We then shared two examples from the scientific literature to demonstrate how this information gets reported and how it appears on the NHS Medicines and BNF websites. In the second meeting, we only went through one example due to time constraints.

Questions posed to public partners:

Given everything you now know about the information trialists collect:

i. Are the tables and summaries presented in the journal articles and websites acceptable?
ii. Is there any information missing from any of these sources?

## Results

A summary of the discussions and the main topics raised by public partners are presented in the following.

Public partners’ perspectives on:

### Terminology

i. Participants at both meetings indicated a preference for use of ‘side-effects’ to describe adverse events. They thought people would be more familiar with this term. Group 2 felt that this allows for the possibility that a side-effect could be a positive thing.
ii. Both groups felt that the term ‘harm’ indicated a more severe event and would cause alarm. Harm or adverse effect would indicate something ‘bad’ and differed to side-effect which would be an event that caused more of an inconvenience and could include a benefit. Example of use provided in group 1, ‘*taking this drug helps with x, and it can have these side-effects but if you take too many there will be harm*’.
iii. Group 1 thought ‘unintended effects’ sounded like researchers were trying to ‘*dress something up*’ or ‘*hide*’ something. They thought that it might be used in a last chance situation where patients are running out of treatment options, e.g. ‘*it’s a new drug and you will be the first one trying it out and you don’t know what the consequences would be*’, i.e. it would be used for the very early research cycle of a new drug. However, group 2 took a different view and thought 'unintended effects' could indicate unexpected benefits or effects experienced beyond the patient, e.g. impacts on wider family.
iv. Group 1 interpreted the term ‘safety’ as indicating circumstances it is ok to take, for example ‘*this is safe if taken as per instructions*’ or it is likely to come from patient as a question, e.g. ‘*is it safe to take?*’ Similarly, group 2 indicated that they would interpret this as meaning that the intervention is ‘*good to go*’ and would cover the information provided in the prescribing guidelines.

In summary (Table 3), both groups preferred the use of ‘side-effect’ and thought it was an appropriate term to use across all contexts and the use of ‘harm’ should be used to indicate more severe events. Opinion differed for use of ‘unintended effects’ and both groups interpreted ‘safety’ as pertaining to the instructions to ensure safe use of a treatment.

**Table 3:** Feedback on current terminology in use

Current terminology in use	Feedback
Side-effect	Preferred term, appropriate to use across all contexts
Harm	Should be used to indicate more severe events
Unintended consequences	Mixed opinions, instilled mistrust in some and others thought a more encompassing term that covers benefits and events beyond the patient
Safety	Pertains to the instructions to ensure correct use of a treatment

### Communication

#### Communication of results in academic journals

Example structures of the tables of adverse events that were presented at the meetings can be found in Tables 1 and 2. Public partners’ feedback of these tables included:

i. After learning about all the information collected in the original trials (e.g. severity, recurrence, timing and duration of adverse events) and reviewing these example tables, public partners indicated the following:
  - Group 1 felt that all information collected should be reported.
  - Group 2 would want their doctor to have seen any information on recurrence, severity (as it provides an indication on how it can impact their day-to-day life) and duration.
  - Group 1 thought it was important to communicate to patients the probability they will have such events.
ii. Given the impact of data collection methods on the number and type of events that get reported (e.g. spontaneous reports versus prompted reports), group 2 were keen that data collection methods were clearly reported alongside the results. This would give context to the magnitude of frequency of events reported.
iii. Group 1 would prefer that the list of adverse events observed was presented in order of prevalence and not in alphabetical order.
iv. Group 1 thought that the information presented and format was suitable for consumption by researchers but not by the public where they thought visualisations could help.

#### Public facing material

Public partners’ feedback on the public facing material presented included:

i. Group 1 were happy with the simplicity of the information presented in these materials.
ii. Group 2 assumed that the serious events listed on the NHS website were as common as the common events that preceded them, as there was no indication of numbers experiencing serious events. Once this misunderstanding was clarified, they wanted to see how likely the serious events were.
iii. Members of both meetings expressed the need to make public facing material accessible, especially for non-native English speakers. They suggested that this could include analogies to give context to numbers or visualisations. In addition, some felt it would be beneficial for patients to be able to speak to someone who could verbally communicate this information but that this did not need to be a doctor, e.g. the pharmacist would be an acceptable alternative.
iv. Group 1 indicated they were happy not to receive all the information their doctor received, but that they want their doctor to distil to them the important information.
v. Group 2 wanted to be able to assess the trade-off with potential benefits and wanted this information presented alongside the side-effects.
vi. Group 2 wanted to be able to understand what the likely immediate side-effects were and if anything was known about longer-term side-effects. It was noted that information on the timing of events relative to when interventions were first taken was not reported.

### Collection

Group 2 queried whether public involvement groups are ever consulted on how adverse events will be collected in trials. We are not aware of trialists involving the public routinely in adverse event collection methods but public partners thought it was an important area to consult a public involvement group on, as researchers would do for primary or secondary outcomes.

### Reporting

Group 2 felt that historically when they have reported side-effects to their doctor, this information has not been taken seriously, for example they have been told ‘*not to worry*’. Public partners in group 2 were unaware of the ability to self-report side-effects via the MHRA’s Yellow Card scheme; this topic was not raised in group 1 discussions.

## Discussion

Despite the importance of considering potential harmful intervention effects in medical decision-making, our understanding of patient and the public’s views on the communication of this information in clinical trials is limited. In this article, we describe public involvement activities that sought public opinion on terminology used to describe and communicate harmful effects.

### Main findings (summarised in Table 4)

**Table 4:** Summary of main discussion points raised by our public partners

1	Whilst researchers have sought an agreement for a term for monitoring the potential harmful effects of interventions, public partners endorse the use of different terms for different situations.
2	Public partners preferred the use of ‘side-effect’ and thought it was an appropriate term to use across all contexts and the use of ‘harm’ should be used to indicate more severe events.
3	Public partners would like clinical staff to have complete information on the potential harmful effects of interventions from clinical trials including information on the likely severity and duration of adverse events; the relevant information should then be distilled appropriately to patients.
4	Generally, the public partners were happy with the type of information presented in public facing materials but discussions revealed the lack of information presented in some areas (e.g. frequency of serious events on the NHS website) could lead to misconceptions.
5	Public facing materials should be accessible, especially for non-native English speakers, with visualisations being proposed as one solution by public partners.
6	The public facing material does not allow for an easy trade-off of benefits to side-effects and lacks information on the likely timing of any side-effects relative to treatment initiation.
7	Public partners wanted to understand what the likely immediate side-effects were and if anything was known about longer-term side-effects.

There was a clear steer from our public partners that they preferred the use of the term ‘side-effects’, reserving the use of ‘harm’ for more severe events. This will require researchers to work with patients to find a suitable distinction between side-effects and harms going forward when communicating results. Our public partners reported that they do not need to see the same information as healthcare providers, who they believe should have all the relevant information available to them so that they can distil the necessary information to patients.

Our discussions revealed an important misunderstanding about the information presented on the NHS website. An assumption was made by public partners that the serious events reported (for escitalopram) were as frequent as the common events reported immediately before, as no numbers or qualitative categorisation of frequency (e.g. common or rare) were explicitly reported on the frequency of the serious events. Further investigations across the NHS website showed that frequencies of serious events are inconsistently reported. We do not know how widespread the miscomprehension of this information is by the public, but it highlights an important finding that needs to be addressed in public facing material.

Our public partners wanted to be able to make an easy trade-off between side-effects and benefits when reviewing public facing material. They suggested that the benefits should be presented alongside the side-effects. This reflects recommendations in the literature for patient decision aids (PtDAs) from 2013 that indicates ‘*side-by-side presentation of information and options in PtDAs is associated with increased perception of balance*’ and is in line with the 2022 work from Bruhn et al. where participants ‘*expressed a need to judge for themselves whether treatment benefit outweighed negative side effects*’ [17, 18]. They also raised the importance of ensuring these materials were accessible to a range of audiences, suggesting visualisation had a part to play. Again, in line with the literature on best practice for communicating risk to patients [17, 19]. Finally, they wanted information on timing to be presented to be able to distinguish between likely immediate side-effects and longer-term side-effects. This highlights an acknowledged limitation with trial data which is likely to represent only a short period of follow-up. It also revealed an important omission; the timing of events relative to treatment initiation was missing across all presentations reviewed [19]. There is much to be learnt from the existing literature on improving the presentation of potential harmful intervention effects in public facing materials.

### Our findings in context of what is already known

Safety in the context of clinical trials is the ‘*the avoidance, prevention, or mitigation of harms or hazards that arise from the use of medicinal products*’ [20]. Talk of an intervention’s ‘safety profile’ is really describing how unsafe it is. The 2004 CONSORT harm extension promoted the use of the term ‘harms’ over the use of ‘safety’, which they felt was a ‘*reassuring term*’ that could ‘*obscure the real and potentially major “harms” that drugs and other interventions may cause*’ [7]. This was further supported by the recent CONSORT harm update (2022) with the authors continuing to recommend that the term safety be avoided, stating that ‘*the term “safe” might give the impression that harms are not caused by an intervention or could imply that the trialists or the sponsoring drug company judged that the potential benefits of the intervention assessed outweighed the potential harms (at least under the trial conditions)*’ [9]. Contrary to our discussions they also recommended avoidance of the use of ‘side-effect’, as it ‘*denotes an effect without identifying it as a harmful one*’, and this was supported by patient representatives who reported that ‘*side-effect downplays harm*’. The authors do not report the number or characteristics of the patient representatives that contributed to the Delphi survey where these results were obtained so we are unable to comment on likely reasons for these differences in language preference.

Areas for improvement highlighted in discussions with our public partners closely align with existing good practice guidance on communication. For example, the Trevena et al. guidance on risk communication recommends presenting simple frequencies such as 1 in 100, keeping the denominator consistent. However, the NHS website uses the language of ‘1 in x’, varying the denominator across events, which is reported to be ‘*more difficult to understand and elevate[s] risk perceptions*’ [19].

To allow patients the opportunity to weigh up the potential benefits of a treatment with their potential harm, we believe there are some key lessons to be learnt from the Winton Centre on communication of risk, for example their work on the COVID-19 vaccine using a graphic to present the risk of developing a blood clot following vaccination [21, 22]. The graphic illustrated the potential benefit in terms of ICU admissions prevented due to vaccination against the risk of a specific type of blood clot thought to be related to the vaccine. Whilst this image was not without its limitations as commented on by the authors, it is ‘*limited to [a] fixed time but benefits and harm could accrue over lifetime of vaccines protection, and ICU admissions and blood clots not only events of interest*’, it did receive widespread endorsement, ‘*many people found this choice of comparison (and visualisation) helpful and thought it provided an appropriate context for the numbers*’ [22]. Despite the availability of this image, the NHS website continues to list COVID-19 side-effects separate to the benefits, failing to capitalise on this communication tool designed specifically to present benefit alongside harm. Adaptations of this work beyond COVID-19 vaccines present an opportunity to improve risk communication of medicines to the public, in line with public partners’ feedback and already established good practice principles.

### Strengths and limitations

We varied the location of the meeting allowing input from geographically diverse groups. Due to wide interest in this meeting, we were also able to ensure participants from a diverse range of ages, ethnicities, education and employment backgrounds were consulted. Replication of many of the key discussion points across the meetings also adds weight to these perspectives. However, as we were only able to host two meetings, these discussions might be limited in their generalisability.

We were also limited in the number of examples we could share and discuss in the meetings due to time constraints, but from our previous work we were able to choose examples that were an accurate representation of how this information is typically communicated in journal articles and public facing materials, which use a standard format across interventions [10, 11]. The examples used in the meetings should not have limited the scope of discussions.

Our discussions did not cover the communication of potential adverse events in trial recruitment material; however, this has previously been discussed in Svobodova et al. who propose seven principles to improve the communication of potential treatment benefits and harms in participant information sheets [23]. We also did not discuss the communication of adverse events in the dissemination of trial results to trial participants; this has been highlighted as a priority by Bruhn et al. for future research [18].

### Future research

Further research on how trial results are communicated to trial participants and the wider public would be beneficial. In addition, a key priority for future work should be to ensure clarity and accessibility of public facing material.

## Conclusions

Our understanding of the public’s views on the communication of adverse events in clinical trials is limited but this work identifies key areas to engage with public partners on in clinical trials in the future. It provides a starting point on preferred terminology. Whilst researchers have tried to seek agreement for a term for monitoring the potential harmful effects of interventions, public partners endorsed use of different terms for different situations. It also highlights some key areas for improvement in public facing materials that are necessary to avoid miscommunication and the potential for an incorrect perception of harm.

## Data Availability

All data and materials are included in this publication.
